# Comparison of Iris-Claw Phakic Lens Implant versus Corneal Laser Techniques in High Myopia: A Five-Year Follow-Up Study

**DOI:** 10.3390/healthcare10101904

**Published:** 2022-09-28

**Authors:** Gracia Castro-Luna, Noelia Sánchez-Liñán, Hazem Alaskar, Antonio Pérez-Rueda, Bruno José Nievas-Soriano

**Affiliations:** 1 Department of Nursing, Physiotherapy, and Medicine, University of Almería, 04009 Almería, Spain; 2 Department of Ophthalmology, Poniente Hospital, 04700 Almería, Spain; 3 Department of Ophthalmology, University Torrecardenas Hospital, 04009 Almería, Spain

**Keywords:** iris-claw, phakic lens implant, corneal laser, high myopia, Femto-LASIK, PRK, Artiflex, Artisan

## Abstract

Background. This study aimed to compare the efficacy and safety of Femto-LASIK, PRK, and Artiflex/Artisan phakic lens implantation in the surgical correction of myopia at different moments of postoperative follow-up; to propose a linear predictive model of visual acuity without correction at five years of refractive procedures; and to evaluate its validity. Methods. A retrospective observational analysis was performed. Patients were clinically reviewed after three months, one year, two years, and five years. Univariate and bivariate analyses and a multivariate linear regression model were performed. Results. Six hundred seventy-nine eyes were analyzed: 18.9% Artiflex, 2.8% Artisan, 42.3% Femto-LASIK, and 36.1% PRK. There were significant differences in effectiveness and safety after five years when comparing Artiflex/Artisan versus PRK and Femto-LASIK (*p* < 0.01). The linear regression model explained 30.32% of the patients’ visual acuity variability after five years. Conclusions. PRK surgery, Femto-Lasik, and Artiflex/Artisan type phakic lens implantation are effective, safe, and predictable techniques with stable refractive results. Phakic lenses magnify myopic patients who improve their UCVA and BCVA. Concerning phakic lens implantation, corneal endothelial cells remain stable. The predictive model calculated that surgery with a phakic lens increased the UCVA result at five years, and surgery with PRK slightly decreased the long-term results.

## 1. Introduction

According to the World Health Organization (WHO), myopia, in general, is defined as a refractive error with a spherical equivalent (SE) equal to or greater than 0.50 diopters in each eye. High myopia is defined when the SE equals or exceeds 6.00 diopters in each eye [1,2,3]. This threshold was also defined by the American Academy of Ophthalmology [4].

The concept of high myopia should not be confused with pathological myopia. Although the excessive elongation of the eye and the presence of a posterior staphyloma could be promoted factors in the development of degenerative changes associated with the latter [5], refractive error or axial length are not criteria “per se” of pathological myopia [6,7]. Pathological myopia can also be defined as an entity in which chorioretinal atrophy is equal to or more severe than diffuse atrophy [7,8]. In Western Europe, according to some authors, the percentage of myopic people in 2020 will be around 30–35% [9]. The increase in the number of patients with high myopia [9] leads to an increase in cataracts [10], glaucoma, retinal detachment [11], or pathologic myopia [7].

Treating refractive errors, especially myopia, has been one of the fastest-growing fields of ophthalmology in recent decades. These surgical procedures allow the patient to eliminate his dependence on glasses. However, they do not prevent the appearance of the problems mentioned due to myopic condition. Currently, refractive surgery techniques can be simplistically classified into techniques based on applying an excimer laser on the cornea with three types. These techniques are excimer laser application on the corneal surface (PRK) and techniques of excimer laser application before a full flap either with a femtosecond laser (Femto-LASIK), with a mechanical microkeratome (LASIK) or incomplete flap (SMILE). The other techniques are based on implanting an intraocular lens in the anterior chamber (Artisan/Artiflex) or in the posterior chamber (ICL). Intraocular lens implantation is usually prescribed when the patient has contraindicated corneal surgery with an excimer laser or the number of diopters exceeds the recommended number of diopters with laser techniques.

Nowadays, it has avoided ablations of large areas that increase the risk of postoperative corneal ectasia and the presence of optical aberrations that limit the patient’s final visual outcome. Faced with these limitations, implanting a phakic lens to correct refractive errors appears to be an option.

Among the phakic lenses, angular-supported phakic lenses have practically disappeared from the market due to the frequent association with a decrease in the endothelial cell population in the medium and long term [12,13]. Refractive surgeons use the Implantable Posterior Camera Lens (ICL) or the iridian fixation phakic lens as Artiflex and Artisan. Artiflex is a foldable lens that fixes the position of the iris and the anterior chamber through an incision of 3.2 mm and has only a single size in its diameter, unlike other phakic lenses. Artisan is the equivalent unfoldable model for more than −14 diopters of myopia. The anatomical requirements for implanting both lenses are similar [14,15,16,17,18,19,20,21].

Therefore, this work aims to compare the efficacy and safety of Femto-LASIK, PRK, and Artiflex/Artisan phakic lens implantation in the surgical correction of myopia at three months, one, two, and five years of evolution; to propose a linear predictive model of visual acuity without correction at five years of refractive procedures; and to evaluate its validity.

## 2. Materials and Methods

### 2.1. Study Design and Patient Selection

A retrospective observational analysis was performed. The data source was the medical records database of the patients treated at the Medical Vision Institute, located in Almeria, southeast Spain.

The inclusion criteria were not wearing contact lenses two weeks before surgery, stable refraction at least two years before surgery, and age over 21; in the case of corneal surgery: corneal topographic stability and sufficient pachymetry according to the refractive defect to be corrected. In addition, in the case of phakic lenses, the anterior chamber depth is greater than or equal to 3.4 mm as measured from the epithelium. Endothelial cell counts greater than or equal to 2500 cells/mm^2^, mesopic pupil diameter (under low light) less than or equal to 6.5 mm, and astigmatism less than or equal to 2.00 D.

The general exclusion criteria for any type of refractive surgery were patients under 21 years of age, active eye pathology, cataract, glaucoma (in case of phakic lenses), chronic recurrent uveitis, previous eye surgery, macular or retinal pathology, systemic autoimmune disease, diabetes mellitus, and pregnancy. Further exclusions for PRK and Femto-LASIK were cases with evidence of ectasia or suspicion of keratoconus evidenced by corneal topography estimated postoperative corneal thickness was less than 350 microns, ocular disease, or active systemic disease affecting corneal healing. The study does not include retreatment cases for any refractive surgery.

### 2.2. Data Collection

Patients were clinically reviewed at the center at three months, one year, two years, and five years after the surgical procedure. All patients were high myopes (spherical equivalent greater than six diopters). For the phakic lens implantation technique, 147 patient eyes were analyzed, all operated on by the same surgeon. The lens implanted in all cases was the folding phakic Worst model with iridian fixation. The anterior chamber location (Artiflex, Ophtec, Groningen, The Netherlands) was used to correct myopia from −6 to −14 diopters. The criteria for choosing PRK or Femto-LASIK were topographic stability, preoperative pachymetry, and calculated ablation depth. In addition, phakic lens surgery was proposed in all cases of laser surgery contraindications.

The preoperative examination included: Uncorrected Visual Acuity (UCVA) and Best-Corrected Visual Acuity (BCVA) (Topcon ACP8 Optotype Projector) with the Snellen type of letters and using the decimal scale, from 0.05 to 1 in mesopic conditions(combination of photopic and scotopic vision under low-light (but not necessarily dark) conditions.). Contact lens wearers were asked to stop wearing contact lenses two weeks before the examination. The ophthalmologic variables were obtained with the following equipment: the refractive measurements were obtained using the auto refractometer–keratometer model Nidek (ARK-700, Nagoya, Japan), the eye biomicroscopy with the Slit-lamp model HaagStrait (BQ 900, Berna, Switzerland) was performed to rule out the presence of pathology in the anterior ocular pole that contraindicated surgery. The intraocular pressure was measured by non-contact tonometry (Reichert Inc., Buffalo, NY, USA). The pachymetry was obtained by ultrasonic pachymetry (DGH 500. DGH Technology Inc., Exton, PA, USA). The axial logarithm length of the eyeball was determined by ultrasonic biometry (DGH 500. DGH Technology Inc., Exton, PA, USA). The funduscopy was performed with the pole lens model Superfield (NC. Volk Inc., Mentor, OH, USA) and the indirect ophthalmoscopy with a +20 D lens (Volk Inc., Mentor, OH, USA). The corneal topography examination was carried out using a projection corneal topography using a Placido disc, obtaining an elevation map, and an aberrometry study of the anterior face of the cornea (CSO, Florence, Italy). The specular endothelial cell microscope (SP-2000, Topcon, Japan) was used to obtain a photographic image of the endothelium using corneal reflection. The calculation of cells is performed automatically with polygonization of 20 cells marked manually on the image taken. Finally, the pupillary diameter was determined under mesopic conditions (Pupilographer, Florence, Italy).

### 2.3. Description of the Surgical Techniques

Regarding surgical techniques and Excimer Laser, three days before surgery, the patient was prescribed cleaning of the eyelids with Cilclar wipes (Alcon), treatment with diclofenac sodium drops 0.1% (Voltaren, Novartis AG, Basilea, Switzerland), and Ofloxacin eye drops 3 mg/mL (Exocin, Allergan Inc, Irvine, CA, USA). Then, the surgery was performed using topical anesthetic drops, oxybuprocaine 0.4%, and tetracaine. All surgeries were performed by the same surgeon, following the same technique and protocol [22]. The excimer laser used in all cases was the OSIRIS Laser (OSIRIS, SCHWIND, Kleinostheim, Germany). Laser calibration was performed at the beginning of each surgery.

In the case of PRK, the de-epithelialization was performed with the laser according to the described technique [22]. Diluted mitomycin C 0.02% was used during PRK surgery for at least 20 s. Postoperative treatment was tobramycin eye drops (Tobrex, Alcon Laboratories, Ft Worth, TX, USA) three times daily and 0.25% fluorometholone (FML Forte, Allergan Inc., Irvine, CA, USA) prescribed four times daily for one month. The haze or regression was treated with topical corticosteroids when necessary.

In the case of Femto-LASIK, a superior hinged flap of 8.5–9 mm in diameter and thickness was made with the femtosecond laser (Intralase, Abbot), depending on the patient. The depth of the keratectomy ranges from 90 to 400 microns [23]. Lamellar dissection is achieved by minimal impacts, around 3 microns in diameter. The impacts are applied following a grid pattern. Subsequently, the flap must be lifted with a blunt spatula, starting in an area close to the hinge. Postoperative treatment was tobramycin and dexamethasone (Tobradex, Alcon Laboratories, Ft Worth, TX, USA) four times a day for one week.

The lens implantation procedure was the same for all cases and was performed following these steps: One week before surgery, an upper iridotomy was performed with a YAG laser (Nidek, Tokyo, Japan) to prevent a possible blockage in the circulation of the aqueous humor. Intraoperative miosis was maintained by perfusion of acetylcholine in the anterior chamber (Acetylcholine 10 mg/mL Cusí, Lab. Alcon). Two 1.5 mm lumbar incisions are made at III h and IX h. The anterior chamber was maintained by injecting Artivisc viscoelastic 0.55 mL (Lab. Ophtec, Groningen, The Netherlands). A 3.2 mm limbal incision is made at XII h, through which the lens is inserted into the anterior chamber using the insertion spatula provided. The lens is oriented on the iris in the chosen position and locked into the iris tissue underlying the haptics, using specific holding and locking forceps [24,25]. Then, 0.1 mL of cefuroxime 1% is introduced into the anterior chamber to prevent endophthalmitis. Postoperative treatment was tobramycin and dexamethasone (Tobradex, Alcon Laboratories, Ft Worth, TX, USA) four times a day for the first week and a weekly descending pattern for up to 4 weeks.

### 2.4. Definition of Effectiveness and Safety Indexes

The efficacy index is defined as the ratio of postoperative UCVA to preoperative BCVA for each period. This ratio measures whether the patient achieves an uncorrected postoperative vision similar to pre-surgery vision with the spectacle prescription.

The safety index is the ratio of postoperative BCVA to preoperative BCVA for each patient in each follow-up period. This ratio measures whether the patient achieves postoperative corrected vision similar to pre-surgery vision with the best spectacle prescription.

### 2.5. Statistical Analyses and Review Board Approval

SPSS version 27 (IBM Inc., Armonk, NY, USA) and R statistical software version 3.5.1 (R Foundation for Statistical Computing, Vienna, Austria) were used in the statistical analysis. The data were expressed with the mean and standard deviation (SD) for quantitative variables and frequencies and percentages for qualitative variables. The Kolmogorov–Smirnoff test was used to check the normality of the quantitative variables. *p* values of less than 0.05 in this test indicated that the variables did not follow a normal distribution in some time intervals, so it was necessary to apply non-parametric tests (Mann–Whitney U). The period variable was analyzed two by two in the bivariate analysis with the Wilcoxon test. Differences were considered statistically significant for an alpha error of less than 0.05 (*p* < 0.005).

A multivariate linear regression model was calculated. The dependent variable was the UCVA at five years. All the requirements of the multivariate linear regression model were reviewed: the linear relationship between the dependent variable and the independent quantitative variables (graph of aggregate variables), the absence of collinearity between variables (IVF < 2.5), homoscedasticity (homogeneity of the variance of the model calculated by Breusch–Pagan test), normality of the residuals of the model verified by the Shapiro–Wilk test.

The principles of the Declaration of Helsinki of the World Medical Association were followed. In addition, all patients signed an Informed Consent form in advance of surgery more than 24 h before surgery and were provided with a copy. The patients signed to authorize the surgery and the use of data for research purposes. This research was approved by the Ethics Committee of Nursing, Physiotherapy, and Medicine Department of the University of Almeria (Spain), with reference number 179/2022. The authors declare no commercial interest or any conflict of interests.

## 3. Results

The study included 245 eyes of 191 high myopic patients treated with the PRK technique, 287 eyes of 171 patients treated with the Femto-LASIK technique, and 147 phakic lenses of 95 patients implanted between 2010 and 2011. The distribution of the total number of 679 eyes by surgical technique was: Artiflex (128 eyes, 18.9%), Artisan (19 eyes, 2.8%), Femto-LASIK (287 eyes, 42.3%), and PRK (245 eyes, 36.1%).

### 3.1. Descriptives and Comparisons between Surgical Techniques

Descriptives and comparisons of efficacy between surgical techniques are shown in Table 1 and Table 2, respectively.

Descriptives and comparisons of safety between surgical techniques are shown in Table 3 and Table 4, respectively.

### 3.2. Linear Regression Model

A linear regression model whose dependent variable was the UCVA at five years was established. After analyzing the statistical significance between the preoperative and the dependent variables, a linear model was calculated. The surgical technique was classified into the phakic lens, PRK, and FS-LASIK. The model was calculated using the forward-backward variable inclusion and exclusion method. Table 5 shows the statistical significance of the linear regression model coefficients.

The model explained 30.32% of the variability of the patients’ visual acuity at five years.

The algorithm would be expressed as follows:

UCVA 5 years = 0.96 + 0.04 (Sph Equiv preop) + 0.43 (Phakic lens) − 0.08 (PRK).

The model predicts that surgery with the phakic lens increased the UCVA result (0.43 more) at five years, and surgery with PRK (−0.08) decreased this result.

### 3.3. Complications

**Figure 1:**
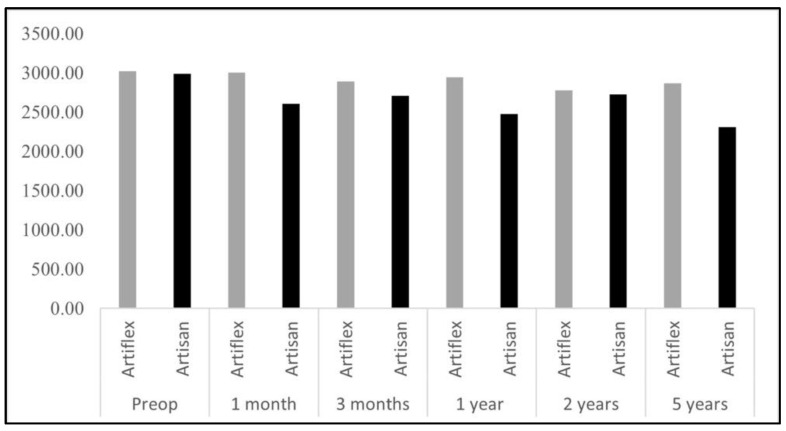
Endothelial cells accounts mean.

There were no severe complications in refractive surgery with laser and phakic lenses. After Artiflex phakic lens surgery, the corneal endothelial cells remained stable during the follow-up period, although there was a moderate decrease in the patient’s preoperative status. The endothelial cell count decreased significantly in the Artisan implant, although it remained above 2000 cells per mm^2^ (Figure 1).

## 4. Discussion

The main aim of this research was to compare the efficacy and safety of Femto-LASIK, PRK, and Artiflex/Artisan phakic lens implantation in the surgical correction of myopia and to propose a linear predictive model of visual acuity without correction at five years of refractive procedures and to evaluate its validity.

This study has determined that the mean safety index of all techniques at five years was more significant than one (Table 2). However, the efficacy index at five years of the surgical techniques (Table 1) was 0.82 and 0.86 for PRK and FS-LASIK corneal ablation techniques, respectively, and higher for phakic lenses. In addition, there is a statistically significant difference between the 5-year effectiveness of PRK and Femto-LASIK with Artiflex lens implantation, with the efficacy of the lens being superior (Table 3).

Gershoni et al. [26] reported that the clinical outcomes of Femto-LASIK were slightly better than those of PRK. Another study compared the results of Femto-LASIK and PRK to correct high myopia and found that Femto-LASIK showed that UCVA was better than PRK [27]. Hashemi et al. [28], in a 6-month follow-up, found efficacy rates of 0.99 ± 0.07 and 0.93 ± 0.22 (*p* = 0.192) in Femto-LASIK and PRK, respectively, and safety rates of 1.01 ± 0.05 and 1.01 ± 0.14 (*p* = 0.949), respectively. Hersh et al. [29], in a prospective randomized multicenter study with a 6-month follow-up, concluded that although the improvement in uncorrected visual acuity is faster in LASIK than in PRK, the long-term efficacy and safety results are generally similar between the two procedures in the correction of moderate-high myopia. Sorkin et al. [30] demonstrated that high myopia PRK with mitomycin-C application in eyes at risk of developing high ectasia is a safe and effective alternative to LASIK. In a systematic review and meta-analysis, Wen et al. [31] showed no statistically significant differences in visual outcomes in efficacy and safety between Femto-LASIK and PRK. Femto-LASIK performed better in predictability than PRK.

The mean safety has been above 1 in all follow-up periods in all phakic lenses. The evolution of efficacy has been above one throughout the follow-up period, reaching a maximum of 1.16 at two years and decreasing slightly to 1.10. Comparatively, studies have published efficacies one year after surgery with an index of 1.13. The studies referring to Artisan refer to efficacy indices at one year between 0.79 and 1 [32,33]. Cakir et al. [34], in a review of 5-year results, concluded that Artisan IOL implantation is an effective and safe procedure for the surgical treatment of high myopia. A similar conclusion is drawn in Monteiro et al. [35] and Charters et al. [36], referring that phakic intraocular lenses are extremely useful in high myopia and an excellent addition to refractive armamentarium in clinical practice. Hashemi et al. [37], in their comparison study between PRK-MMC and phakic lens implantation, show that phakic IOL implantation was better than PRK-MMC in correcting high myopia in terms of visual quality. However, the two methods had no difference in visual acuity. According to the Miraftab et al.’s [38] 3-year results, phakic lens implantation is better than PRK-MMC for treating patients with myopia > 8.0 D. A systematic review by Wu et al. [39] compared both types of iris-anchored phakic lenses, rigid and foldable, provided updated evidence. They found that the foldable lens group was superior in efficacy and safety in treating high myopia to the rigid lens group. Yuan et al. [40], after a 5-year follow-up, showed that lens implantation fixed to the anterior iris was effective, predictable, and reversible in correcting high myopia in phakic eyes.

Other authors [41] conclude that the phakic lenses are the first choice in correcting high ametropia and in cases where the ocular surface or cornea is not suitable for keratorefractive techniques, and the excellent results of safety and efficacy that are obtained are confirmed. After three years of follow-up, Morral et al. [42] show that the Artisan iridian fixation phakic IOL is an effective and safe procedure for correcting moderate-severe refractive errors.

This research has some limitations. The main limitation of this work is the sample size, and as in most prospective studies, many patients are lost to follow-up at five years for unknown reasons and do not allow the possibility of complications to be identified. Another limitation is the potential selection bias, as all the patients were chosen from the same center. These aspects should be considered when assessing the external validity of our findings. For future research, it could be helpful to perform multicentric studies with larger sample size.

## 5. Conclusions

PRK surgery, Femto-Lasik, and Artiflex/Artisan type phakic lens implantation are effective, safe, and predictable techniques after three months and one, two, and five years, with stable refractive results throughout the follow-up periods. Phakic lenses magnify myopic patients who improve their UCVA and BCVA superior to their preoperative conditions. Concerning phakic lens implantation, corneal endothelial cells remain stable during the follow-up period, although there is a moderate decrease in the patient’s preoperative status. The predictive model calculated that surgery with a phakic lens increased the UCVA result at five years, and surgery with PRK slightly decreased the long-term UCVA result.

## Figures and Tables

**Table 1:** Descriptive statistics efficacy index according to techniques.

		N	Mean	SD *	95% Confidence Interval	
					Lower Limit	Upper Limit
Efficacy 1 month	PRK	245	0.69	0.35	0.65	0.73
	FS-Lasik	284	0.84	0.38	0.79	0.88
	Artiflex	70	1.03	0.59	0.89	1.18
	Artisan	5	0.88	0.49	0.28	1.48
Efficacy 3 months	PRK	243	0.83	0.36	0.78	0.88
	FS-Lasik	273	0.95	0.38	0.91	1
	Artiflex	71	1.07	0.48	0.94	1.19
	Artisan	6	0.97	0.45	0.50	1.44
Efficacy 1 year	PRK	236	0.86	0.33	0.82	0.91
	FS-Lasik	253	1.00	0.69	0.91	1.08
	Artiflex	53	1.15	0.44	1.02	1.27
	Artisan	8	1.30	0.35	1.01	1.59
Efficacy 2 year	PRK	229	0.91	0.35	0.87	0.96
	FS-Lasik	249	0.93	0.41	0.88	0.98
	Artiflex	24	1.16	0.37	1.00	1.31
	Artisan	4	0.91	0.56	0.01	1.81
Efficacy 5 year	PRK	241	0.82	0.42	0.77	0.87
	FS-Lasik	252	0.86	0.46	0.81	0.92
	Artiflex	35	1.10	0.24	1.01	1.18
	Artisan	2	1.40	0.10	0.46	2.33

* SD: Standard deviation.

**Table 2:** Comparison between surgeries techniques’ efficacy in the follow-up periods.

			Mean Differences	SD **	*p*-Value
	PRK	FS-Lasik	−0.15 *	0.03	<0.01
		Artiflex	−0.34 *	0.08	<0.01
Efficacy 1 month		Artisan	−0.19	0.22	1.000
	FS-Lasik	Artiflex	−0.19	0.08	0.120
		Artisan	−0.04	0.22	1.000
	Artiflex	Artisan	0.15	0.23	1.000
	PRK	FS-Lasik	−0.12 *	0.03	<0.01
		Artiflex	−0.24 *	0.06	<0.01
Efficacy 3 months		Artisan	−0.14	0.18	1.000
	FS-Lasik	Artiflex	−0.11	0.06	0.560
		Artisan	−0.02	0.18	1.000
	Artiflex	Artisan	0.09	0.19	1.000
	PRK	FS-Lasik	−0.13	0.05	0.060
		Artiflex	−0.28 *	0.07	<0.01
		Artisan	−0.43	0.13	0.090
Efficacy 1 year	Fs-Lasik	Artiflex	−0.15	0.08	0.410
		Artisan	−0.30	0.13	0.390
	Artiflex	Artisan	−0.15	0.14	0.970
	PRK	FS-Lasik	−0.04	0.04	0.960
		Artiflex	−0.28 *	0.05	<0.01
Efficacy 5 year		Artisan	−0.58	0.08	0.410
	FS-Lasik	Artiflex	−0.23 *	0.05	<0.01
		Artisan	−0.53	0.08	0.430
	Artiflex	Artisan	−0.30	0.09	0.580

* *p* < 0.05 ** SD: Standard deviation.

**Table 3:** Descriptive statistics Safety index according to techniques.

		Mean	SD *	Standard Error	95% Confidence Interval	
					Lower Limit	Upper Limit
Safety 1 month	PRK	0.90	0.32	0.02	0.86	0.94
	FS-Lasik	1.09	0.34	0.02	1.05	1.13
	Artiflex	1.24	0.55	0.08	1.08	1.41
	Artisan	1.58	0.19	0.11	1.11	2.05
	Total	1.03	0.37	0.02	1.00	1.06
Safety 3 months	PRK	1.03	0.35	0.02	0.98	1.07
	FS-Lasik	1.16	0.38	0.02	1.11	1.20
	Artiflex	1.25	0.58	0.09	1.07	1.44
	Artisan	1.46	0.24	0.11	1.16	1.76
	Total	1.11	0.39	0.02	1.08	1.14
Safety 1 year	PRK	1.07	0.36	0.02	1.02	1.11
	FS-Lasik	1.22	0.67	0.04	1.14	1.30
	Artiflex	1.14	0.20	0.04	1.06	1.23
	Artisan	1.19	0.36	0.12	0.91	1.47
	Total	1.15	0.53	0.02	1.10	1.19
Safety 2 year	PRK	1.10	0,4	0.03	1.05	1.15
	FS-Lasik	1.20	0.44	0.03	1.14	1.25
	Artiflex	1.14	0.20	0.05	1.03	1.24
	Artisan	1.37	0.14	0.06	1.20	1.54
	Total	1.15	0.42	0.02	1.12	1.19
Safety 5 year	PRK	1.14	0.39	0.02	1.10	1.19
	FS-Lasik	1.24	0.50	0.03	1.18	1.30
	Artiflex	1.1	0.30	0.09	0.90	1.30
	Artisan	1.24	0.22	0.13	0.68	1.79
	Total	1.19	0.45	0.02	1.16	1.23

* SD: Standard deviation.

**Table 4:** Mean differences between surgeries techniques safety in the follow-up periods.

			Mean Differences	SD *	*p*-Value
	PRK	FS-Lasik	−0.18	0.03	<0.01
		Artiflex	−0.34	0.09	<0.01
		Artisan	−0.68	0.11	0.200
Safety 1 month	FS-Lasik	Artiflex	−0.16	0.09	0.530
		Artisan	−0.49	0.11	0.350
	Artiflex	Artisan	−0.34	0.14	0.460
	PRK	FS-Lasik	−0.13	0.03	<0.01
		Artiflex	−0.23	0.10	0.200
		Artisan	−0.44	0.11	0.140
Safety 3 months	FS-Lasik	Artiflex	−0.10	0.10	0.980
		Artisan	−0.30	0.11	0.390
	Artiflex	Artisan	−0.21	0.14	0.850
	PRK	FS-Lasik	−0.15	0.05	0.020
		Artiflex	−0.08	0.05	0.670
		Artisan	−0.12	0.12	0.990
Safety 1 year	FS-Lasik	Artiflex	0.08	0.06	0.880
		Artisan	0.03	0.13	1.000
	Artiflex	Artisan	−0.04	0.13	1.000
	PRK	FS-Lasik	−0.09	0.04	0.150
		Artiflex	0.04	0.09	1.000
		Artisan	−0.09	0.13	1.000
Safety 5 year	FS-Lasik	Artiflex	0.14	0.10	0.850
		Artisan	0.00	0.13	1.000
	Artiflex	Artisan	−0.14	0.16	1.000

* SD: Standard deviation.

**Table 5:** Coefficients of the linear regression model.

	Coefficient	SD	t Value	*p*-Value
Constant	0.96	0.05	20.476	<0.001 *
Sph Equival Preop	0.04	0.01	10.716	<0.001 *
Reference Technique = Femto-LASIK				
Technique = Phakic Lens	0.43	0.04	10.316	<0.001 *
Technique = PRK	−0.08	0.02	−3.069	<0.001 *

* Dependent Variable: UCVA 5 years postop. R^2^: 0.3071.R^2^ adjusted: 0.3032. *p*-value: < 0.001.

## Data Availability

All data are contained within the article.
